# Antibiotics increase aggression behavior and aggression-related pheromones and receptors in *Drosophila melanogaster*

**DOI:** 10.1016/j.isci.2022.104371

**Published:** 2022-05-07

**Authors:** M. Grinberg, R. Levin, H. Neuman, O. Ziv, S. Turjeman, Gila Gamliel, R. Nosenko, O. Koren

**Affiliations:** 1 Azrieli Faculty of Medicine, Bar Ilan University, Safed, Israel

**Keywords:** behavioral neuroscience, microbiome

## Abstract

Aggression is a behavior common in most species; it is controlled by internal and external drivers, including hormones, environmental cues, and social interactions, and underlying pathways are understood in a broad range of species. To date, though, effects of gut microbiota on aggression in the context of gut-brain communication and social behavior have not been completely elucidated. We examine how manipulation of *Drosophila melanogaster* microbiota affects aggression as well as the pathways that underlie the behavior in this species. Male flies treated with antibiotics exhibited significantly more aggressive behaviors. Furthermore, they had higher levels of cVA and (Z)-9 Tricosene, pheromones associated with aggression in flies, as well as higher expression of the relevant pheromone receptors and transporters OR67d, OR83b, GR32a, and LUSH. These findings suggest that aggressive behavior is, at least in part, mediated by bacterial species in flies.

## Highlights

• Aggression increases in flies that lack a microbiome
• Monocolonization with specific bacteria can mediate this effect
• We observed differences in aggression-related pheromone expression levels

## Graphical abstract

**Figure:**
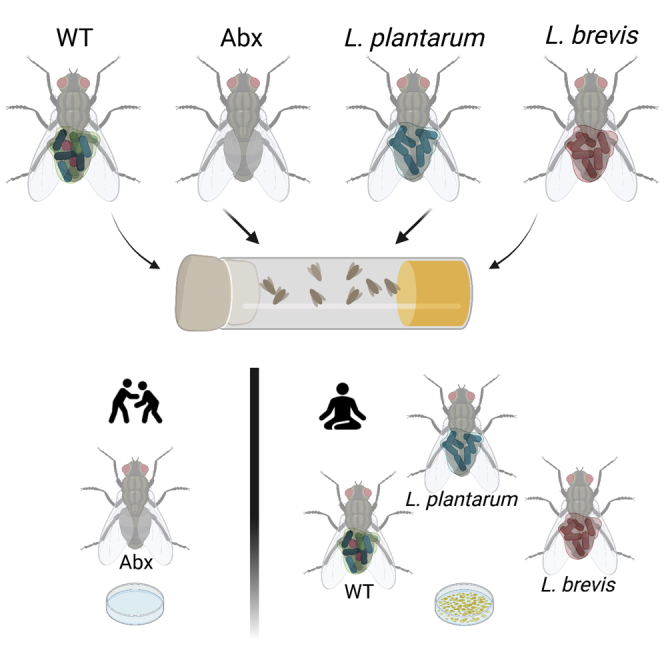

## Introduction

Aggression is evident in almost all animal species and can be influenced by specific genes, neurotransmitters, neural systems, pheromones, hormones, social interactions, and other environmental factors (Edwards et al., 2009a, 2009b; Fischer et al., 2017). Aggression and pathways controlling it are well studied in model organisms (Aleyasin et al., 2018; Golden et al., 2019; Kim et al., 2017; Nassel and Zandawala, 2019; Thomas et al., 2015), but a nuanced understanding of how certain biological processes interact with these pathways is lacking. Specifically, it is evident that the gut microbiota can greatly influence aspects of host physiology, including gut–brain communication and social behavior (Agranyoni et al., 2021; Nicholson et al., 2012; Sharon et al., 2010; Sherwin et al., 2019), but to date, the effect of the gut microbiota on aggression and underlying pathways is not fully understood. In the current study, we asked whether the microbiome plays a role in aggression and if so, what pathways may be involved.

Here we focus on *Drosophila melanogaster* because aggression has been well-studied in this simple animal model (Kravitz and Fernandez, 2015). The neuronal mechanisms leading to aggression in *D. melanogaster* have been identified and mainly include pheromones and olfactory sensory neurons that express odorant receptors (Tirindelli et al., 2009); the relevant neuromodulators are extensively reviewed by Asahina (2017). Furthermore, previous studies of gut-brain-behavior interactions in this species demonstrated a clear influence of antibiotics on mating preference, correlated with alterations in cVA levels (Cortot et al., 2022; Sharon et al., 2010); cVA is a male-specific pheromone known to affect courtship and aggression in fruit flies (Ejima, 2015). In addition, the gut endosymbiont *Wolbachia* was shown to alter pheromone production in *D. melanogaster* pupae, interfering with their communication and causing gamete incompatibility (Pontier and Schweisguth, 2015). Because of its relatively simple microbiota composition and the ability to easily manipulate it to test the effects of single bacterial species on behavior, *D. melanogaster* is currently one of the preferred model animals in the field of gut–brain communication and social behavior (Leitao-Goncalves et al., 2017; Peng et al., 2008; Sharon et al., 2010; Wong et al., 2011). Two major members of the *Drosophila* microbiome are *Lactobacillus plantarum* and *Lactobacillus brevis*. These bacteria have many roles, most of them related to maintaining overall homeostasis and fly growth (Park et al., 2015). Moreover, *L. plantarum* has a unique role in hormonal growth signaling and promotes growth even under poor nutrition, probably by targeting rapamycin (TOR) activation (Storelli et al., 2011).

Previous studies have confirmed that the microbiota plays a role in gut–brain communication and subsequent behavior (Fischer et al., 2017; Nicholson et al., 2012; Sharon et al., 2010). Research specifically related to aggression is limited, but there is evidence of microbiota differences between aggressive and non-aggressive dogs (Kirchoff et al., 2019). Studies examining effects of antibiotics or germ-free conditions on host aggression in flies (Jia et al., 2021), mice (Watanabe et al., 2021), and hamsters (Sylvia et al., 2017) provide contradicting results, but there is evidence that *Wolbachia* infection in flies can increase aggression (Rohrscheib et al., 2015). Accordingly, we hypothesized that alterations in the fly microbiome would affect male aggression behavior by modulating expression of the related pheromones (cVA and (Z)-9 Tricosene) (Ejima et al., 2007; Sengupta and Smith, 2014; Tirindelli et al., 2009) and receptor and transporter components (OR67d, OR83b, GR32a, and LUSH) (Fleischer et al., 2018; Liu et al., 2011; Xu et al., 2005). Through a set of manipulations, we studied how specific changes to microbial composition alter aggressive behavior and examined how the microbiota interacts with relevant pheromones and receptors.

## Results

### Changes in the microbiome alter aggression in *D. melanogaster*

To test our overarching hypothesis that microbiome alterations affect male fly aggression, we measured aggression (Edwards et al., 2009b) in four experimental groups of male *D. melanogaster*: (1) **untreated** flies (control group, “cmy-WT” for cornmeal, molassess, yeast-wild type), (2) **Abx** flies grown on media supplemented with a mixture of antibiotics (to eliminate gut bacteria), (3) ***L. brevis***-monocolonized flies, and (4) ***L. plantarum***-monocolonized flies. The flies in groups 3 and 4 were offspring of flies grown on antibiotics that were transferred to sterile media supplemented with the focal microbe. Research in our lab supports that growth media in generation F1, rather than parental microbiome (or F0 media-type), drives the F1 microbiota composition.

**Figure 1:**
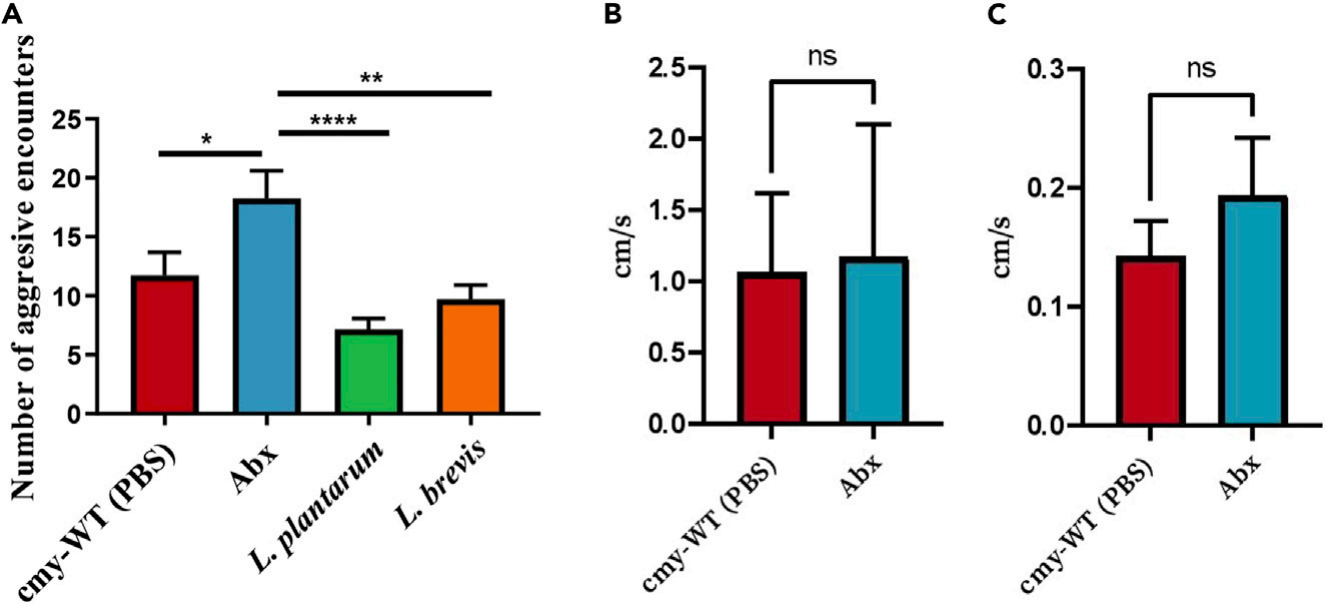
Aggression levels are influenced by microbial changes in *D. melanogaster* (A) Behavior tests showing the different number of aggressive encounters in the four treatment groups. The Abx treated flies showed higher levels of aggression than any other treatment, while treatment with a mono-culture of *L. plantarum* proved to reduce the aggression levels most significantly (n = 20 with eight male flies vials in each treatment). Statistical tests were calculated by one-way ANOVA∗< 0.05 ∗∗ < 0.005 ∗∗∗∗<0.0001. (B and C) Average distance moved per time tracked for each fly for a subset of videos filmed in the light or dark period. There is no difference between the groups in the daylight (B) or at night (C). Statistics were performed based on the average distance moved per time tracked (as defined using Ethovision v16). Videos were chosen blindly to capture morning, afternoon, evening, and night. Behaviors of six flies in each experimental group were included in the analysis. n.s= not significant. the bars indicate S.E.. cmy-WT is the untreated group.

To examine aggressive behavior it is necessary to examine group interaction, as it is not always possible to identify aggression in one-on-one interactions, potentially because there are not enough aggression pheromones to cause the flies to perform behaviors defined as aggressive (Zwarts et al., 2012). We found that Abx treatment increased the number of aggressive encounters among male flies compared to the control group by nearly 150% (Figure 1; p-value ∗< 0.05) whereas supplementation with a single bacterial species (*L. plantarum* or *L. brevis*) reduced aggression compared to both the Abx-treated flies (Figure 1A; significant: p-value ∗∗∗∗<0.0001and p-value ∗∗<0.005, respectively) and the control group (marginally significant, p-value = 0.09). These results validated our hypothesis that bacteria can modulate aggression. To ensure that fly hyperactivity did not confound findings regarding aggression, we filmed flies for 24 h and selected videos covering morning, afternoon, evening and nighttime stretches. Using EthoVision XT v16 (Noldus; Wageningen, the Netherlands), we quantified total distance traveled for the flies in videos filmed under light and dark conditions. There were no differences between the Abx and cmy-WT groups (n = 6 flies per treatment) in either of the light conditions (Figures 1B and 1C; Mann-Whitney U tests, p > 0.05).

### Levels of pheromones change according to the microbiome composition

**Figure 2:**
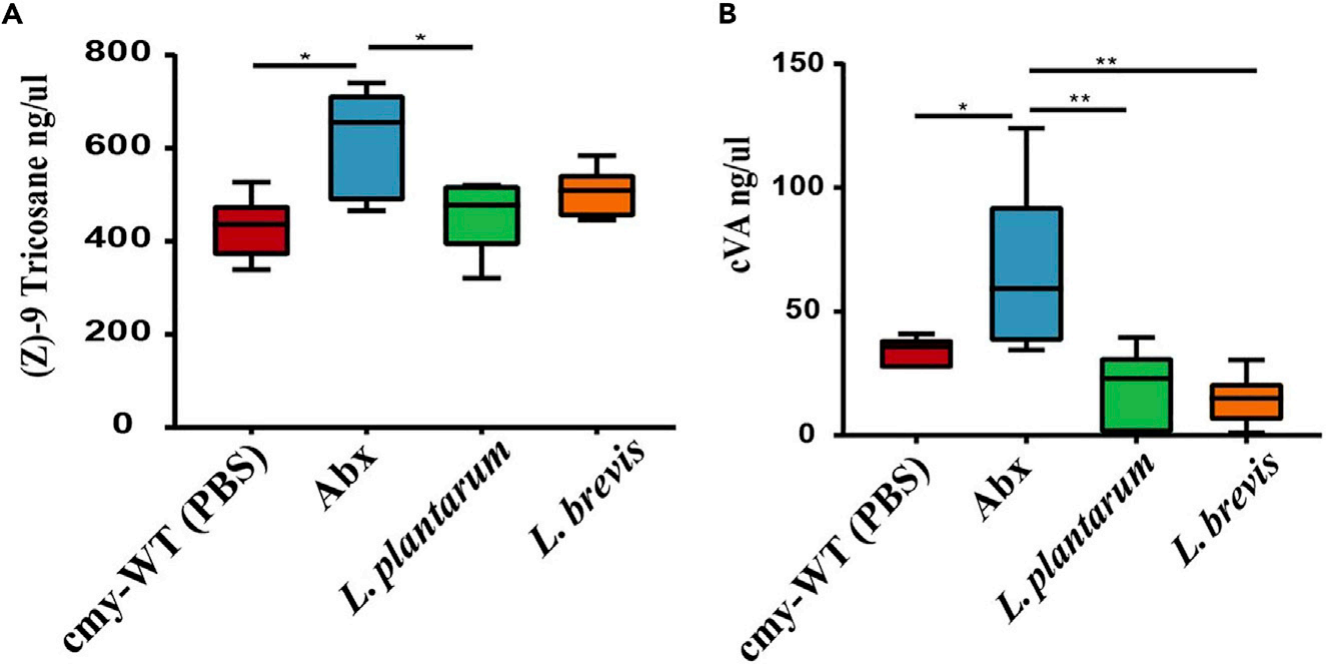
Levels of pheromone changes according to the microbiome composition (A) Levels of aggressive pheromones in male flies. Pheromone levels calculated by concentration of internal standard with GC-MS (n = 8 vials with eight male flies in each treatment) (A) Expression of (Z)-9 Tricosane levels by treatment. Higher levels measured in Abx treated flies and lower levels in *L. plantarum*. (B) Expression of cVA levels by treatment. Higher levels measured in Abx treated flies and lower levels in *L. plantarum* and *L. brevis*. Statistical tests: all graphs calculated by one-way ANOVA∗< 0.05 ∗∗ < 0.005 ∗∗∗∗<0.0001. The bars indicate S.E. cmy-WT is the untreated group.

To decipher the mechanism underlying this interaction, we first examined how the gut microbiota influences levels of cVA and (Z)-9 Tricosene (9-T), pheromones typically positively associated with aggressive behavior in male fruit flies (Wang and Anderson, 2010). Using the same experimental set up, we found that levels of both pheromones were significantly higher in Abx-treated flies than in other treatment groups (Figures 2A and 2B); specifically, cVA and 9-T levels were on average 2 times greater than the control, respectively.

### Changes in the bacterial composition affect expression of genes associated with aggression

**Figure 3:**
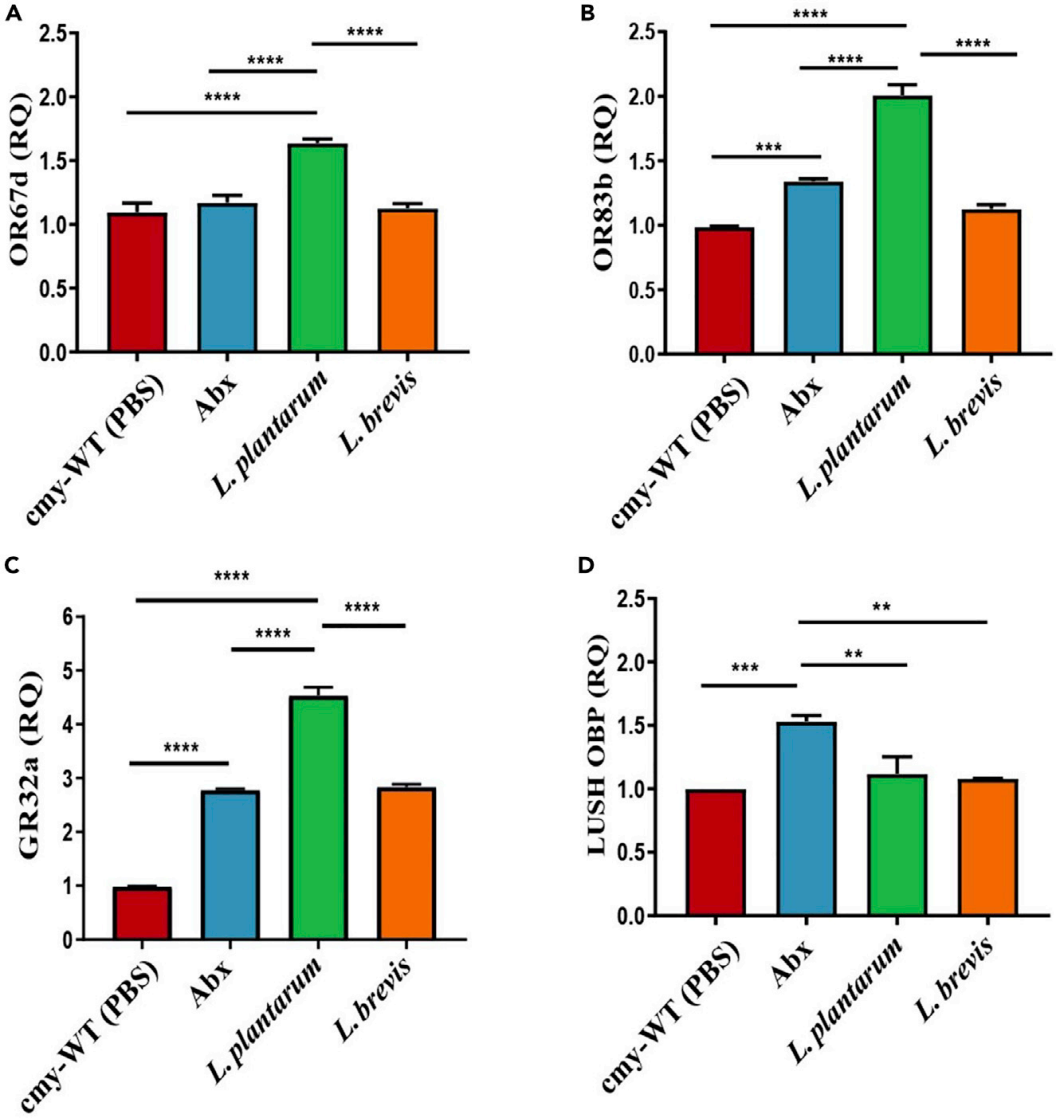
RNA expression of OSN in the different groups Levels of RNA were calculated by using qPCR and obtaining the RQ value in each group n = 30 male flies (3 biological repetitions). (A) OR67d. (B) OR83b (ORCO). (C) In *L. plantarum* treated flies the expression was higher than other treatments in GR32a. (D) Higher levels were observed in Abx treatment in LUSH OBP expression. Statistical tests on all graphs were calculated by one-way ANOVA∗∗< 0.005 ∗∗∗ < 0.001 ∗∗∗∗<0.0001. The bars indicate S.E. cmy-WT is the untreated group.

We next examined the effect of microbiota on three pheromone receptors associated with aggression, OR67d and OR83b, receptors of cVA (Ejima et al., 2007), and GR32a, a receptor of 9-T (Wang et al., 2011), using qRT-PCR to quantify their expression (Figures 3A–3C). Abx treatment significantly raised levels of OR83b and GR32a, but not OR67d, compared to untreated flies. Interestingly, *L. plantarum* supplementation significantly and most dramatically raised levels of all three receptors whereas *L. brevis* supplementation resulted in receptor levels comparable to or slightly greater than untreated flies. In addition to measurements of receptor expression, we also compared levels of the cVA transporter LUSH between groups (Xu et al., 2005) (Figure 3D). Again, the Abx treatment resulted in significantly higher expression levels compared to all other groups.

## Discussion

We tested the hypothesis that fruit fly microbiota affects male aggression with behavioral tests as well as experiments that examined pheromone production and receptor/transporter expression. This hierarchical study design helped us to identify the cascade of effects that the microbiota has on the physiology of aggression by approaching the pathway holistically. Our findings show that Abx treatment increased aggression in male flies, as compared to untreated flies. This finding is in direct contrast to those recently published by Jia et al. (2021) who studied aggression in germ-free (GF) flies and found reduced aggression in flies with depleted microbiota. The procedure by which GF flies were produced, which may have a range of consequences on the host that interacts with those related to the microbiota, should be taken into account. Further, facility-related effects on the WT fly microbiomes and differences in behavioral assays may also explain these findings.

In addition to our findings of increased aggression, Abx-flies produced higher levels of aggression pheromones cVA and (Z)-9 Tricosene and exhibited higher expression of the related receptor components OR83b and GR32a and cVA transporter LUSH. This finding is in line with a preprint reporting a slight but significant increase in global cVA in WT adult male flies hatched from dechorionated eggs (without a microbiome at hatching) compared to normally hatched WT flies (Cortot et al., 2022). Taking all of our results together, we conclude that the natural gut microbiota in the fly plays a role in regulating male aggression by both modulating pheromone production as well as expression of their receptors and associated proteins.

Of note, while Abx treatment significantly raised levels of OR83b and GR32a, as compared to untreated flies, it did not seem to influence OR67d, suggesting that this may be a less important receptor of cVA than OR83b. Also of interest, in the treatment group supplemented with *L. plantarum*, we found low levels of aggression accompanied by low pheromone levels, yet receptor levels were significantly higher than in all other groups. This phenomenon might be explained by a negative feedback loop previously described in this pathway (Benton, 2007; Fleischer et al., 2018). Further research in a knock-out model or using pheromone manipulation can help to identify the mechanisms underlying observed changes in aggression on the whole and also the puzzling interaction effect of Abx and bacterial supplementation on receptor levels.

## Conclusions

Our study is one of the first to show the relationship between antibiotics, aggression, and also pheromones and receptor levels. Understanding these relationships can provide more information about gut-brain communication necessary for deciphering behavioral mechanisms related to aggression as well as additional behaviors. Further monocolonization studies can uncover the nuances associated with this behavior, and bacterial species found to moderate aggression can be examined for similar interactions in other species.

### Limitations of the study

In this article, we used only one strain of fly stocks, Oregon R. Future studies could use additional aggression assays, for example, studying flies in pairs, or examine germ-free flies without effects of antibiotics.

## STAR★Methods

### Key resources table

**Table:** 

REAGENT or RESOURCE	SOURCE	IDENTIFIER
**Bacterial and virus strains**		

*Lactobacillus plantarum*	Isolated from *Drosophila melanogaster* that were crushed and cultured on MRS	
*Lactobacillus brevis*	Isolated from *Drosophila melanogaster* that were crushed and cultured on MRS	

**Chemicals, peptides, and recombinant proteins**		

Tetracycline	Sigma-Aldrich, St. Louis, Missouri, USA	60-54-8
Rifampicin	Fisher Scientific International Inc, Pittsburgh, Pennsylvania, USA	13292-46-1
Streptomycin	Thermo Fisher Scientific, New Jersey, USA	11860038

**Critical commercial assays**		

5X single RT MasterMix	abm, Vancouver, BC, Canada	discontinued
Total RNA purification kit	Norgen, Thorold, ON, Canada	17200

**Experimental models: Organisms/strains**		

*Drosophila melanogaster* – Oregon R	Bloomington Drosophila Fly Stock Center	5

**Oligonucleotides**		

OR67d F: ATTTTGCGGAAACGATGTGGC R: GGATTATGGTGAGGTCTCCATTG	Sengupta and Smith, 2014	
OR83b F: TCACGAAGTTTATCTACCTGGCT R: ATCGAATGGTAACGAGCATCC	Sengupta and Smith, 2014	
GR32a F: CTATGAGGTGGGTCCTCCGA R: CGTCTCGCGGTAGGAGAAAA	Sengupta and Smith, 2014	
LUSH F: GACCTCGCTAGACATGATCCG R: GCACATAAGATCCTGCGATGG	Sengupta and Smith, 2014	

**Software and algorithms**		

GraphPad Prism version 8 for Windows	GraphPad Software, San Diego, California USAs	
EthoVision XT version 16	Noldus, Wageningen, the Netherlands	

### Resource availability

#### Lead contact

Further information and requests for resources, reagents and strains should be directed to and will be fulfilled by the lead contact, Omry Koren (omry.koren@biu.ac.il).

#### Material availability

Fly stocks (Oregon R) were obtained from Bloomington Drosophila Stock Center (Indiana Avenue, Bloomington, IN, USA).

*L. plantarum* or *L. brevis* were isolated in house from *Drosophila melanogaster* that were crushed and cultured on MRS.

This study did not generate new unique reagents.

### Experimental model and subject details

#### Fly stocks

Fly stocks (Oregon R) were obtained from Bloomington Drosophila Stock Center (Indiana Avenue, Bloomington, IN, USA). Flies were reared in 50 mL vials (10 cm long, 2.5 cm diameter) containing 10 mL CMY (cornmeal, molasses, yeast) growth media. Flies were maintained in an incubator at 25°C with a light dark cycle of 12 h:12 h. We used male flies that were 4–7 days old for all experiments and analyses.

#### Antibiotic treatment (Abx)

An antibiotic mixture containing three types of antibiotics (50 μg/mL tetracycline, 200 μg/mL rifampicin, and 100 μg/mL streptomycin) was added to CMY media. In order to functionally test the effectiveness of the Abx supplementation, a PCR reaction using microbial primers for the 16S rRNA gene (515F+806R) (Caporaso et al., 2012) was performed and showed absence of microbial DNA.

#### Single microbe supplementation

Abx-treated flies were transferred to new vials containing CMY media (vials were sterilized prior to treatment in UV light for 15 min) supplemented with 100 μL of an overnight culture (∼10^8^ bacteria) of either *L. plantarum* or *L. brevis* diluted in sterile PBS. The bacterial concentration chosen is comparable to levels in our untreated flies. Offspring of transferred flies (second generation) were used for experiments.

### Method details

#### Aggression experiment

Eight male flies aged 4–7 days were collected in empty vials and starved for 2 h (Wang and Anderson, 2010). The eight flies (Zwarts et al., 2012) were then transferred (without anesthesia) to an empty vial containing a patch of yeast-water (the size of a micro-spatula, 1.4 mg/mL water) and a decapitated female, which provide ideal conditions for aggression (Wang and Anderson, 2010). For the first 5 min, the flies were left to adapt to the new vial. Their activity was then recorded for 5 min using Panasonic HC-V550 (720p) and HC-V770 (1080p) video cameras with default frame rate. The total number of aggression encounters within the vial was recorded. For the purpose of counting movements, scoring was done by dividing the vial into the upper half and the lower half and then counting each incidence of a visible movement and summing the total for the two halves. Focal movements were either lunging, boxing, chasing, or wing threats. At least 20 aggression tests (20 separate vials of eight flies) were analyzed per experimental group. All tests from each group are presented in the graphs; the error bars represent the standard error of the mean of the number of movements observed in each vial. Scoring was performed in a blinded manner. The experiment was carried out over several weeks. On each day of testing, vials with flies from all four groups were included to ensure no group-specific bias resulting from experiment day. Further, all tests were conducted at the same time of day on each of the experimental days. To ensure that fly hyperactivity did not confound findings regarding aggression, we filmed flies that underwent the same treatments and that were kept alone, one fly per tube for 24 h. We selected videos covering morning, afternoon, evening and nighttime stretches and quantified total distance traveled for the flies in videos filmed under light and dark conditions using EthoVision XT v16 (Noldus; Wageningen, the Netherlands).

#### Gas chromatography analysis

Eight flies aged 4–7 days were separated into an empty glass vial and starved for 2 h. Pheromones were then extracted from fly cuticles by adding 1,000 μL hexane for 5 min at room temperature. The liquid was transferred to a GC-MS adjusted vial, and 10 μL of hexane containing 2000 ng/μL of hexocosane (C-26) was added as an internal standard. Vials were shaken for 1 min. Extracts were concentrated to 10 μL, of which 2 μL were injected into a HP-5/mS silica capillary column (30 m∗0.25 mm∗0.25 mm film thickness) that was temperature-programmed: 140°C (2 min), 3°Cmin^-1^ to 300°C (2 min). Extracts were analyzed by gas chromatography coupled with mass spectrometry (Clarus SQ 8T GC/ Mass Spectrometer, Perkin Elmer, Walthman, MA, USA). The NIST mass-spectral library identifications were confirmed with chemical standards when available (Sigma-Aldrich). Compounds of interest (cVA and (Z)-9 Tricosene) were identified based on their mass spectrum and retention time and quantified by peak integration. These two compounds were chosen because they have previously been identified as pheromones associated with aggression in male flies (Wang and Anderson, 2010).

#### qRT-PCR

For each treatment group, ten male flies (4–7 days old) were collected and anesthetized. Decapitation of heads was performed using sterile tweezers. RNA was purified following homogenization of the heads with a total RNA purification kit according to the manufacturer’s protocol (NORGEN, Thorold, ON, Canada). The first strand of cDNA (see protein targets below) was synthesized from 5X single RT MasterMix (abm, Vancouver, BC, Canada) using reverse transcriptase. Quantitative real-time PCR was performed using the StepOne^TM^ Real-Time PCR System (Thermo Fisher Scientific, Waltham, MA, USA). Reactions included a mixture of 5 μL 2X SYBR, 1 μL of each primer (10 μM; see below), and 4 μL cDNA per sample. In the negative control, cDNA was replaced with DDW. Primers were designed using Primer-BLAST (NCBI) and FlyPrimerBank (DRSC; https://www.flyrnai.org/FlyPrimerBank) for well-studied pheromone receptors and a transporter as targets: OR67d (receptor for vCA), OR83b (vcA), GR32a (9-T), and LUSH (transporter for vCA) (Sengupta and Smith, 2014).

OR67d F: ATTTTGCGGAAACGATGTGGCR: GGATTATGGTGAGGTCTCCATTG

OR83b F: TCACGAAGTTTATCTACCTGGCTR: ATCGAATGGTAACGAGCATCC

GR32a F: CTATGAGGTGGGTCCTCCGAR: CGTCTCGCGGTAGGAGAAAA

LUSH F: GACCTCGCTAGACATGATCCGR: GCACATAAGATCCTGCGATGG

### Quantification and statistical analysis

Statistical analyses were performed in GraphPad Prism version eight for Windows (GraphPad Software, San Diego, California USA, www.graphpad.com). Two-tailed one-way ANOVAs were used to test differences in aggressive encounters and pheromone and related protein expression levels among the four experimental treatments, followed by Tukey tests, when appropriate. For fly hyperactivity, we used EthoVision XT v16 to quantify distance measures and then compared them in Prism using Mann-Whitney U tests. Throughout, ∗< 0.05 ∗∗ < 0.005 ∗∗∗<0.001 ∗∗∗∗<0.0001.

## Data Availability

All data reported in this paper will be shared by the lead contact upon request. No codes were generated for this study.
